# Iron in seeds – loading pathways and subcellular localization

**DOI:** 10.3389/fpls.2013.00535

**Published:** 2014-01-02

**Authors:** Louis Grillet, Stéphane Mari, Wolfgang Schmidt

**Affiliations:** ^1^Institute of Plant and Microbial BiologyAcademia Sinica, Taipei, Taiwan; ^2^Plant Biology, Institut National pour la Recherche AgronomiqueMontpellier, France

**Keywords:** biofortification, grain filling, Fe transport, Fe storage, Fe in seeds

## Abstract

Iron (Fe) is one of the most abundant elements on earth, but its limited bioavailability poses a major constraint for agriculture and constitutes a serious problem in human health. Due to an improved understanding of the mechanisms that control Fe homeostasis in plants, major advances toward engineering biofortified crops have been made during the past decade. Examples of successful biofortification strategies are, however, still scarce and the process of Fe loading into seeds is far from being well understood in most crop species. In particular in grains where the embryo represents the main storage compartment such as legumes, increasing the seed Fe content remains a challenging task. This review aims at placing the recently identified actors in Fe transport into the unsolved puzzle of grain filling, taking the differences of Fe distribution between various species into consideration. We summarize the current knowledge on Fe transport between symplasmic and apoplasmic compartments, and provide models for Fe trafficking and localization in different seed types that may help to develop high seed Fe germplasms.

## INTRODUCTION

Iron (Fe) is involved in the transport of electrons in many ubiquitous metabolic processes such as respiration and photosynthesis, and is required as a co-factor of numerous enzymes. Although highly abundant in the earth’s crust, the low solubility of Fe often limits plant growth. This is largely due to the high reactivity of Fe toward oxygen: in soils, Fe tends to form highly insoluble ferric hydroxides, dramatically restricting the bioavailability of Fe. Undernourishment for Fe decreases productivity and yield, posing a major constraint for both agriculture and human health. Among the essential micronutrients, Fe is considered as the most deleterious when present in insufficient amounts (Stoltzfus, 2003; Zimmermann and Hurrell, 2007). In plant-based diets, Fe is provided as non-heme Fe that is less well absorbed than heme-bound Fe in meat, causing Fe deficiency-induced anemia (FDA) in areas where monotonous, plant-based diets are dominating (USA National Institute of Health, Office of Dietary Supplements, http://ods.od.nih.gov/factsheets/Iron-HealthProfessional/). Combating FDA requires strategies to increase the Fe content in crops and, as a prerequisite for the development of such strategies, a better understanding of the mechanisms that control transport of Fe to and storage of Fe in edible plant tissues, as well as the chemical forms in which Fe is present in these tissues.

Efforts toward understanding Fe uptake have been mainly focused on roots (Eide et al., 1996; Robinson et al., 1999; Curie et al., 2001; Nozoye et al., 2011). Plants are traditionally separated into two strategies by which they acquire Fe from the soil (Römheld and Marschner, 1983). Strategy I plants, which include all plants except grasses, acquire Fe after reduction of Fe^III^ chelates by a plasma membrane (PM)-bound ferric chelate reductase (FRO2 in *Arabidopsis*; Robinson et al., 1999); the resulting Fe^II^ is then taken up by a transporter of the ZIP family (Iron-Regulated Transporter1, IRT1, in *Arabidopsis*; Eide et al., 1996; Vert et al., 2002). The solubility of Fe is increased by the P-type ATPase-mediated proton excretion (AHA2 in *Arabidopsis*; Santi and Schmidt, 2009), which decreases the rhizosphere pH and increases pFe in the soil solution. Graminaceous plants take up Fe by secreting plant-borne chelators (phytosiderophores) of the mugineic acid family with high affinity to Fe^III^ via TOM1 (Nozoye et al., 2011), and the Fe^III^-phytosiderophore complex is taken up by a YELLOW STRIPE/YELLOW STRIPE1-like (YSL) family transporter without prior reduction (Curie et al., 2001). Recent findings have faded the border between the two strategies. Iron binding compounds of the coumarin and flavin families have been identified in root exudates of *Arabidopsis* and *Medicago truncatula* (Fourcroy et al., 2013; Rodriguez-Celma et al., 2013). Similar to phytosiderophores, these compounds mobilize non-bioavailable Fe. Secretion of phenolic compounds was also observed in the strategy II plant rice (Bashir et al., 2011; Ishimaru et al., 2011). Another deviation from the initial strategy I/strategy II concept is the presence of a functional Fe^2^^+^ transporter (OsIRT1) in rice roots (Ishimaru et al., 2006).

The flow of Fe through the plant involves the Fe chelators nicotianamine (NA), citrate, and deoxymugineic acid (DMA), which act as chaperones to avoid precipitation and cellular damage by the formation of harmful reactive oxygen species through Fenton chemistry, as well as proteins capable of transporting either these molecules as such or their Fe chelates (Le Jean et al., 2005; Waters et al., 2006; Durrett et al., 2007; Inoue et al., 2009; Rogers et al., 2009; Yokosho et al., 2009; Nishiyama et al., 2011). Ultimately, Fe has to be transported to the places of highest demand, the photosynthetic electron transport chains in leaves, the reproductive organs (Roschzttardtz et al., 2013), and to the seeds where Fe is stored to support embryogenesis. Despite the importance for human nutrition, the latter process is poorly understood. In this review, we are aiming at providing an update on the mechanisms that transports Fe from roots to the seed and to emphasize the current knowledge gaps in the framework of Fe transport to the seeds. We will focus on the proteins involved in this process as well as on the chemical forms of Fe they transport. Finally, we will also discuss the differences in Fe localization between seeds of various plant species and their consequences in terms of chemical speciation and nutritional properties.

## IRON TRANSPORT IN THE SHOOT AND UPTAKE OF Fe INTO AERIAL PLANT PARTS

**FIGURE 1:**
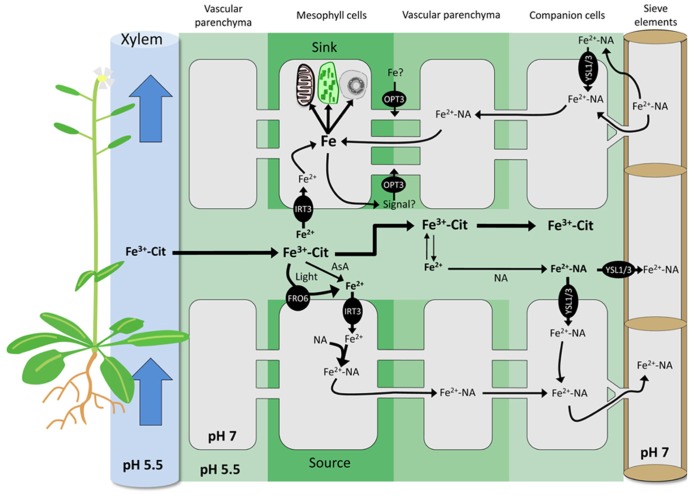
**Exchange of Fe between apoplasmic and symplasmic compartments in *Arabidopsis*.** Root Fe is translocated to shoots through the xylem as Fe^III^-citrate complexes by bulk flow. Fe^III^ can be reduced by light, extracellular AsA, and PM-bound ferric reductases of the FRO family. AtFRO6 and AtIRT3 are likely candidates to mediate Fe reduction and uptake in *Arabidopsis* shoots in both sink and source cells. In the cytoplasm, highly reactive Fe^II^ is complexed by NA. In the apoplasm, Fe^II^ is oxidized to Fe^III^ and chelated by citrate. The formation of Fe-NA complexes in the apoplasm may occur to some extent. Apoplasmic Fe-NA can be taken up by PM-localized YSL transporters. In the cytoplasm, the prevalent Fe form is Fe-NA, which can readily circulate through the symplasmic path. Fe-NA moves from cell-to-cell through plasmodesmata passively by bulk flow. In the phloem, Fe is presumably mainly present as Fe-NA. In sink organs, Fe-NA can exit the phloem via plasmodesmata, or diffuse out of sieve elements into the apoplasm. In the latter scenario, Fe is retrieved by YSLs or by AtOPT3. Iron derived both from the apoplasm and from the symplasmic pathway constitutes the intracellular Fe pool. Plants possess yet unknown sensing and signaling mechanisms to regulate the size of this pool. AtOPT3 has been also suggested to transport signaling molecules that could circulate from the shoot to the root through the phloem, thereby negatively regulating root Fe uptake. AsA, ascorbic acid; Cit, citrate; FRO, Ferric Reductase Oxidase; IRT, Iron-Regulated Transporter; NA, nicotianamine; OPT, OligoPeptide Transporter; YSL, Yellow Stripe-Like; PM, plasma membrane.

In contrast to the abundant data on root Fe uptake and its regulation (for reviews see Palmer and Guerinot, 2009; Hindt and Guerinot, 2012; Thomine and Vert, 2013), relatively little is known regarding Fe transport in shoots. Before reaching the chloroplasts and mitochondria where it is highly required, Fe has to be unloaded from the xylem, distributed to the different tissues, and transported across the PM of the sink cells. None of these mechanisms has yet been deciphered, possibly due to difficulties derived from functional redundancy of transporters involved and possible feedback loops to recalibrate Fe homeostasis in these tissues. In dicots, Fe circulates in the xylem as ferric-citrate complexes (Durrett et al., 2007; Rellan-Alvarez et al., 2010). It has been suggested that Fe uptake by shoot cells is achieved through an IRT-like transporter after reduction of Fe in Fe^III^ chelates to Fe^II^ (Jeong and Connolly, 2009; Palmer and Guerinot, 2009). A candidate for the uptake of Fe into leaf cells is the PM-localized transporter AtIRT3. The protein is highly produced in the xylem and in mesophyll cells (Lin et al., 2009), and was shown to mediate Fe uptake in *Arabidopsis* (Shanmugam et al., 2011). The Fe^III^ reduction system that is likely to co-operate with an IRT-type transporter is a ferric-chelate reductase of the FRO family (**Figure 1**). In *Arabidopsis*, the most highly expressed member of this gene family in shoots is *AtFRO6* (Wu et al., 2005; Mukherjee et al., 2006), encoding a protein located on the PM (Jeong et al., 2008). However, *fro6* knock-out lines do not display any phenotype (Jeong and Connolly, 2009), arguing against an essential function of FRO6 in leaf Fe uptake. Other members of the *FRO* family are either not expressed in shoots or localized to intracellular compartments, suggesting other, FRO-independent reduction mechanisms. Although in aerial plant parts photoreduction of Fe^III^ is likely to occur, the extent to which this process contributes to the overall reduction of Fe in leaves remains to be elucidated. Iron in ferric-citrate complexes is particularly sensitive to this process (Bienfait and Scheffers, 1992), resulting in a fast and complete reduction of Fe^III^ accompanied by degradation of citrate and a rise in pH. As Fe^III^-citrate is the predominant form of Fe in the xylem, photoreduction may represent an important component of xylem unloading. Alternatively, Fe^III^ could also be reduced by direct reaction with molecules such as ascorbate (**Figure 1**). Ascorbic acid is present in millimolar concentrations in most tissues and in all cellular compartments including cell walls of both dicots and grasses (Rautenkranz et al., 1994; Foyer and Lelandais, 1996; Conklin et al., 2000). Ascorbic acid is able to reduce Fe^III^-chelates *in vitro* (Grinstead, 1960; Römheld and Marschner, 1983), and its properties to facilitate Fe transport in mammalians are well described (Sayers et al., 1973; Hallberg et al., 1989; Lane and Lawen, 2008). Several studies show a strong negative correlation between Fe and ascorbic acid (AsA) concentration; therefore, Fe^III^ reduction by ascorbic acid is likely crucial for Fe transport in shoots (Zaharieva and Abadía, 2003; Urzica et al., 2012).

## REMOBILIZATION AND DISTRIBUTION OF Fe

Contrary to the uptake of Fe into leaf cells, its distribution within the plants is relatively well documented. A key role in this process is played by the Fe chelator NA. Despite high Fe concentrations in leaves, the NA-deficient tomato mutant *chloronerva* develops Fe-dependent interveinal chlorosis that can be corrected by exogenous application of NA (the “normalizing factor”; Procházka and Scholz, 1984). The phenotype of *chloronerva* plants is caused by defective expression of the gene encoding NA synthase, *NAS* (Ling et al., 1999). In *Arabidopsis*, a similar phenotype was observed in the *nas4x* quadruple mutant, which harbors mutations in all four *Arabidopsis NAS* genes (Klatte et al., 2009). In *nas4x* plants, the flower, and seed Fe content were also affected, indicating a function of NA in intercellular Fe distribution (Klatte et al., 2009; Schuler et al., 2012). NA is a non-proteinogenic amino acid, structurally analogous to and a precursor for phytosiderophores of the DMA family (Noma et al., 1971). Implication of NA in Fe distribution was further demonstrated by functional characterization of the Fe-NA transporter YELLOW STRIPE1 in maize (Schaaf et al., 2004) and other members of this gene family in several species including rice and *Arabidopsis* (Le Jean et al., 2005; Waters et al., 2006; Gendre et al., 2007; Inoue et al., 2009; Ishimaru et al., 2010). In *Arabidopsis*, *AtYSL1*, and *AtYSL3* were shown to be involved in the shoot to seed translocation of Fe; loss-of-function mutants displayed a decrease of both Fe and NA in seeds (Le Jean et al., 2005; Waters et al., 2006). A similar function has been attributed to *OsYSL2* in rice (Ishimaru et al., 2010). To date, there are only two reports on Fe speciation in the phloem sap. The first study was conducted on *Ricinus communis*, revealing the presence of an Fe transport protein (RcITP; Krüger et al., 2002). A later work conducted in rice showed that DMA is the major Fe chelator in the phloem of this species, whereas NA was in fact mainly bound to zinc (Nishiyama et al., 2011).

## PHLOEM LOADING AND UNLOADING OF Fe

In the current view of phloem loading, Fe is bound to NA and transported into the sieve tubes by YSL proteins (Koike et al., 2004; reviewed in Curie et al., 2009). It is, however, unclear whether the Fe transported by YSLs is localized in the cytosol or in the apoplast. The exchange of solutes between the phloem and surrounding tissues occurs either through plasmodesmata, or across the PMs of adjacent companion or vascular parenchyma cells (Lalonde et al., 2003). The later path requires Fe to be present in the cell wall. Fe-NA is unstable at the slightly acidic pH of the cell wall and Fe-citrate is the predominant form of Fe in apoplasmic environment (von Wiren et al., 1999; Rellan-Alvarez et al., 2008). In such a context, formation of the Fe-NA complex would be rate limiting for phloem loading and unloading (**Figure 1**). A major caveat to this scenario is the absence of any characterized PM-bound Fe efflux transporters in plants. In *Arabidopsis*, a homolog of the mammalian Fe exporter ferroportin (FPN), FPN1, was suggested to perform such a function (Morrissey et al., 2009).

Plasmodesmata function is complex and the mechanisms that determine the selectivity of the transported solutes are not clearly defined. So far, no plasmodesma-localized YSL transporter has been identified. Interestingly, such a location was demonstrated for AtOPT3 (Fernandez-Calvino et al., 2011), a member of the oligopeptide transporters (OPT) protein family with Fe transport activity in yeast (Wintz et al., 2003). Disruption of this gene severely affects cellular Fe homeostasis (Stacey et al., 2008); *opt3* knock-down lines (i.e., *opt3-2*) display constitutive overexpression of *IRT1* and *FRO2* in roots, despite of accumulation of high Fe levels in leaves. Members of the OPT family were shown to transport peptides (Lubkowitz et al., 1997; Lubkowitz et al., 1998) and AtOPT3 is thus an unlikely candidate for the transport of free Fe. Potential transport of a Fe-ligand conjugate has not been tested yet. It was hypothesized that AtOPT3 might transport signaling molecules involved in phloem loading and/or Fe sensing (Stacey et al., 2008; García et al., 2013). Iron accumulation in the distal ends of siliques, in the funiculus and in vascular tissues of the seed coat of *opt3-2* plants suggests an impairment of the unloading of Fe from the phloem rather than compromised loading. Whether AtOPT3 functions in the transport of Fe or plays other roles in Fe homeostasis, for example by transporting a signal molecule or a ligand, remains to be elucidated (**Figure 1**).

The existence of mutants with similar phenotypes in other species, such as the pea mutant *degenerative leaves* (*dgl*), which shows a constitutively activated Fe deficiency response in roots despite high Fe concentrations in leaves (Grusak and Pezeshgi, 1996), points to a conserved regulation mechanism of root Fe uptake through shoot-to-root communication. Phloem Fe is likely to be essential in this process (García et al., 2013). Thus, unraveling the loading and unloading processes would provide a comprehensive picture of plant Fe homeostasis which may reveal new targets for breeding biofortified crops.

## CONTRIBUTIONS OF THE XYLEM AND THE PHLOEM TO SEED LOADING OF Fe

Iron loaded into seeds arrives either via xylem vessels or via the sieve tubes of the phloem. Both paths circulate around the seed coat. Nutrients are not directly unloaded into the endosperm (Van Dongen et al., 2003; Stadler et al., 2005), implying the need for an active and selective transport from the integument to the endosperm. The passage from the funiculus to the embryo requires at least two shifts between the symplasmic and apoplasmic path: unloading of Fe from the phloem into the endosperm and transport from the endosperm to the embryo (Patrick and Offler, 2001). Iron delivered via the xylem derives from the uptake of Fe from the rhizosphere; hence, its concentration depends directly on the expression of *IRT1* and *FRO2* (Brown and Chaney, 1971; Blair et al., 2010). This pool of Fe is readily transported as Fe^III^ chelate to the aerial parts (Durrett et al., 2007), where it is taken up by leaf cells and ultimately reaches the seed coat. Thus, xylem Fe contributes directly to both shoot Fe and seed Fe levels. Phloem Fe derives from remobilization of Fe in senescing leaves, likely present as Fe-NA complex. Therefore, the size of the phloem Fe pool is determined on one hand by remobilization mechanisms, i.e., by NA synthesis and by Fe-NA transport via YSL proteins, and on the other hand by the shoot Fe concentration, that was established by the root uptake system during the plant’s life. With this in mind, it is not surprising that xylem Fe was considered as a more important contributor to seed Fe concentration, even though the ratio may greatly vary among species (Hocking and Pate, 1977). In *Arabidopsis*, it was concluded that the xylem provides 60–70% of the total seed Fe content, whereas the remaining 30–40% originates from senescing leaves, most likely via the phloem stream (Waters and Grusak, 2008). Differences between species are likely to exist, as discussed in (Stomph et al., 2009) for the case of xylem discontinuity at the base of cereal seeds. This discontinuity is absent in rice and therefore allows solutes in the xylem to flow through the seed without symplasmic unloading.

## STORAGE OF Fe IN Seeds

Inside seeds, Fe is essential for embryo development (Stacey et al., 2002; Stacey et al., 2008) but might also become toxic at high concentration. Thus, Fe must be transported into embryos and stored in a stable form that can be remobilized during germination. Embryo Fe transport has rarely been addressed experimentally, although this process is highly relevant for both increasing the seed Fe content and to preserve the fitness of the embryo. Only few genes encoding proteins with functions in Fe transport within seeds have been identified. *OsYSL2* encodes a PM Fe-NA transporter and is expressed in various parts of the seed throughout its development (Koike et al., 2004). The role of OsYSL2 in the transport of Fe to the seed was further confirmed using a RNAi line (Ishimaru et al., 2010). Disruption of *AtOPT3* leads to embryo lethality and decreased expression of the gene (as observed in the *opt3-2* mutant) resulted in reduced Fe content of the embryo (Stacey et al., 2008). The substrate of AtOPT3 is unknown. The citrate efflux transporter FRD3 is expressed in the aleurone layer and in the embryo protodermis, and is known to promote Fe nutrition between symplastically disconnected tissues (Roschzttardtz et al., 2011b). For both AtOPT3 and AtFRD3 the precise function in embryo Fe uptake remains to be elucidated.

**FIGURE 2:**
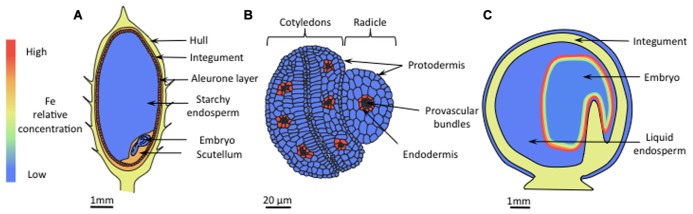
**Iron localization in seeds.** **(A)** In rice, the concentration of Fe is highest in the aleurone layer, the integument, and the scutellum. Iron levels are also high in the hull. In the starchy endosperm the Fe content is low. **(B)** In *Arabidopsis*, Fe is mainly localized in vacuoles of endodermal cells that surround provascular tissues, whereas in other cells the Fe concentration is homogenously low. **(C)** In pea seeds, embryos constitute the main Fe storage pool, but unlike in *Arabidopsis*, Fe is localized in nuclei and plastids. The iron concentration is highest in the outer cell layers, progressively decreasing toward the inner layers.

Development of elemental imaging techniques allowed accurate localization of Fe in seeds of *Arabidopsis,* rice, and pea (Kim et al., 2006; Roschzttardtz et al., 2009, 2011a; Takahashi et al., 2009; Iwai et al., 2012). The results of these studies highlighted major differences between species. In rice seeds, the highest Fe concentration was observed in aleurone layers, integument, and in the scutellum (**Figure 2A**). These tissues are discarded during processing; hence in this species, breeding focused on increasing endosperm Fe content (Goto et al., 1999; Lee et al., 2009) in order to enhance Fe bioavailability (Bashir et al., 2010). In dicots, the endosperm represents a minor portion of mature seeds, and Fe is mainly stored within the embryo. Raising the Fe content in such seeds may therefore be associated with a possible damage of the embryo by toxic Fe concentrations. To prevent such damage, plants possess two mechanisms. Iron can be stored in plastids within ferritins, which assemble as large spherical 24-mer protein complexes able to store up to 4500 Fe atoms in their internal cavities. The second mechanism consists in vacuolar sequestration.

In *Arabidopsis*, during maturation of the embryo Fe is sequestered in vacuoles of the endodermal cell layer (**Figure 2B**) through the VACUOLAR IRON TRANSPORTER1 (AtVIT1; (Kim et al., 2006), and then remobilized during germination by the NATURAL RESISTANCE ASSOCIATED MACROPHAGE PROTEIN3 and 4 (AtNRAMP3 and AtNRAMP4; Lanquar et al., 2005; Roschzttardtz et al., 2009). Quantitatively, it has recently been shown that this particular Fe pool represents around 50% of the total Fe accumulated in *Arabidopsis* embryos (Schnell Ramos et al., 2013). The scenario is quite different in pea embryos where Fe concentration is highest in the epidermis and gradually decreases throughout the inner layers (**Figure 2C**). At the subcellular level, high Fe concentrations have been observed in nuclei and nucleoli although a quantitatively important fraction of Fe is stored in plastids, bound to ferritins (Lobréaux and Briat, 1991). This localization pattern is similar to that observed in leaves of other species (Roschzttardtz et al., 2011a) and young *Arabidopsis* embryos (Roschzttardtz et al., 2009). The diversity in Fe localization reflects different storage forms of Fe. Because of pronounced differences in bioavailability between different forms of Fe, knowledge regarding the Fe ligands is critical for an effective biofortification strategy. Iron-phytate (inositol hexakisphosphate) is an example of non-available Fe (Hallberg et al., 1989). Phytic acids bind tightly to various cations that are not readily released during digestion by mammalians. Phytates are preferentially stored in vacuolar globoids (Wada and Lott, 1997) and constitute the main Fe storage pool of *Arabidopsis* embryos (Lanquar et al., 2005). Recent research has shown that phytate represents also the main Fe storage in the rice aleurone layer (Persson et al., 2009; Iwai et al., 2012). In legume seeds, by contrast, highly bioavailable plastidic ferritin constitutes the main storage form of Fe (Lott et al., 1984; Lobréaux and Briat, 1991; Murray-Kolb et al., 2003; Davila-Hicks et al., 2004). Ferritin was successfully used to engineer rice lines with high seed Fe (Goto et al., 1999; Wirth et al., 2009), indicating that ferritin is a good candidate for biofortification purposes. Although not considered as a natural storage form of Fe, NA was also employed to breed Fe-enriched rice varieties, and manipulating NA content represents so far the most successful attempts regarding to both content and bioavailability of Fe. The first attempt (Masuda et al., 2009) consisted in the expression of the gene encoding barley NA synthase, *HvNAS1*, in rice. Grains of lines overexpressing the NA synthase *OsNAS3* contained around 3-fold more Fe than wild-type plants and were successfully used to heal anemic mice (Lee et al., 2009). Similar results were obtained by overexpressing OsNAS2 (Lee et al., 2012) and *OsNAS1*, specifically in the endosperm (Zheng et al., 2010). In fact, constitutive expression of OsNAS genes was shown to increase the Fe content of polished grains by 2.1- to 4.2-fold (Johnson et al., 2011). Multiple transgene approaches expressing both the ferritin gene from *Phaseolus vulgaris* (*PvFERRITIN*)*,* the *Arabidopsis* NA synthase *AtNAS1*, and the phytase from *Aspergillus fumigatus Afphytase* were successful as well, with a report of a sixfold increase in rice seed Fe content (Wirth et al., 2009). Using a different combination of transgenes (*HvNAS1, OsYSL2*, and *GmFERRITIN*), Masuda et al. (2012) were able to produce field-grown plants with a 4.4-fold increase in Fe content in polished seeds without yield loss. The authors concluded that for efficient biofortification introduction of multiple Fe homeostasis genes is more effective than introduction of single genes.

## CONCLUSION

In the last two decades, understanding of Fe homeostasis in plants has leapt forward dramatically; molecular biology progress led to the identification and functional characterization of many genes and regulatory nodes involved in Fe transport, evolution of analytical techniques has allowed accurate determination of labile Fe species, and, more recently, elemental imaging techniques such as X-ray fluorescence provided new insights into the distribution and trafficking of Fe. Data provided by this array of techniques set the stage for producing Fe-fortified plant varieties, illustrated by the recent achievements in rice (reviewed in Sperotto et al., 2012). Critical gaps in knowledge exist regarding the mechanisms controlling Fe homeostasis in green plant parts, loading and unloading of xylem and phloem, Fe transport within seeds, and, last but not least, regarding the shoot-to-root signal adjusting root Fe uptake to the shoot demand. These questions are pending for decades, complicated by the difficulty to sample phloem sap and intracellular fluids (Kehr and Rep, 2007). Further progress in deciphering the function of several unknown Fe-responsive genes in combination with further technical progress, providing a better resolution of Fe speciation and concentration, will lead to improved strategies to generate Fe-efficient germplasms and to combat FDA.

## NOTES

Since this paper was completed and accepted for publication, the role of ascorbate in iron transport by pea and *Arabidopsis* embryos was unambiguously demonstrated by Grillet et al. (2013).

## Conflict of Interest Statement

The authors declare that the research was conducted in the absence of any commercial or financial relationships that could be construed as a potential conflict of interest.
